# Genome-Wide Association Study for Femoral Neck Bone Geometry

**DOI:** 10.1359/jbmr.090726

**Published:** 2009-07-13

**Authors:** Lan-Juan Zhao, Xiao-Gang Liu, Yao-Zhong Liu, Yong-Jun Liu, Christopher J Papasian, Bao-Yong Sha, Feng Pan, Yan-Fang Guo, Liang Wang, Han Yan, Dong-Hai Xiong, Zi-Hui Tang, Tie-Lin Yang, Xiang-Ding Chen, Yan Guo, Jian Li, Hui Shen, Feng Zhang, Shu-Feng Lei, Robert R Recker, Hong-Wen Deng

**Affiliations:** 1 Osteoporosis Research Center, Creighton University Medical Center Omaha, NE, USA; 2 Laboratory of Molecular and Statistical Genetics, College of Life Sciences Hunan Normal University Changsha, Hunan 410081, Peoples Republic of China; 3 Key Laboratory of Biomedical Information Engineering, Ministry of Education and Institute of Molecular Genetics, School of Life Science and Technology, Xi'an Jiaotong University Xi'an, Shanxi 710049, Peoples Republic of China; 4 School of Medicine, University of Missouri–Kansas City Kansas City, MO, USA

**Keywords:** genome-wide association, femoral neck bone geometry, bone fracture, *RTP3*

## Abstract

Poor femoral neck bone geometry at the femur is an important risk factor for hip fracture. We conducted a genome-wide association study (GWAS) of femoral neck bone geometry, examining approximately 379,000 eligible single-nucleotide polymorphisms (SNPs) in 1000 Caucasians. A common genetic variant, rs7430431 in the receptor transporting protein 3 (*RTP3*) gene, was identified in strong association with the buckling ratio (BR, *P* = 1.6 × 10^−7^), an index of bone structural instability, and with femoral cortical thickness (CT, *P* = 1.9 × 10^−6^). The *RTP3* gene is located in 3p21.31, a region that we found to be linked with CT (LOD = 2.19, *P* = 6.0 × 10^−4^) in 3998 individuals from 434 pedigrees. The replication analyses in 1488 independent Caucasians and 2118 Chinese confirmed the association of rs7430431 to BR and CT (combined *P* = 7.0 × 10^−3^ for BR and *P* = 1.4 × 10^−2^ for CT). In addition, 350 hip fracture patients and 350 healthy control individuals were genotyped to assess the association of the *RTP3* gene with the risk of hip fracture. Significant association between a nearby common SNP, rs10514713 of the *RTP3* gene, and hip fracture (*P* = 1.0 × 10^−3^) was found. Our observations suggest that *RTP3* may be a novel candidate gene for femoral neck bone geometry. © 2010 American Society for Bone and Mineral Research

## Introduction

Osteoporosis is a skeletal disorder that is characterized by low bone strength and an increased risk of bone fracture. In the United States, osteoporosis leads to 5.2 million fractures per decade.(1) Among fractures at various skeletal sites, hip fracture is the most important owing to its high prevalence,(2) high mortality and morbidity,(3) and excessive therapeutic cost.(4,5) The direct cost for hip fracture in the United State was around $13.6 billion in 2005,(6) and this expenditure is expected to increase substantially owing to the population aging. An important risk factor for hip fracture is reduced femoral neck bone geometry at the proximal femur,(7) which is largely determined by low bone mineral density (BMD) and poor femoral neck (FN) bone geometry.(8,9) Prospective determination of FN geometric parameters has significant potential to improve the prediction of hip fracture substantially.(10–12)

FN bone geometry is under strong genetic control.(13–15) Previous whole-genome linkage studies have identified several chromosomal regions linked to FN geometry.(13,15,16) These linkage findings were inconclusive, however, requiring further fine mapping studies because they identified relatively wide genomic regions that typically contain hundreds of genes. Although several candidate genes have been suggested by a small number of association studies of human femoral geometry,(17–21) our current knowledge of genetic factors underlying femoral geometry is relatively superficial.

Genome-wide association study (GWAS) has been used by several research groups to successfully identify genetic factors for a number of complex disorders.(22–29) Currently, this approach has been regarded as one of the most efficient and feasible methods available to locate genes for complex diseases. Recently, Kiel and colleagues(30) conducted a pilot GWAS for FN bone geometry parameters using the Affymetrix 100K SNP GeneChip. Although several interesting regions were identified in that study, some important genes may have escaped detection owing to the relatively low coverage of the 100K Affymetrix GeneChip. To improve our understanding of the genetic basis of femoral bone geometry, we performed a GWAS using highly dense Affymetrix 500K SNP arrays in 1000 Caucasian subjects. We identified a novel gene, *RTP3* (receptor transporting protein 3), associated with two indices of FN bone geometry. The gene's importance to bone strength and osteoporosis was assessed and confirmed by replication in independent Caucasian and Chinese samples.

## Materials and Methods

### Subjects and measurements

The study was approved by the institutional review boards of all involved institutions. Signed informed-consent documents were obtained from all study participants before they entered the study.

We first performed a GWAS (stage 1) in 1000 unrelated Caucasian subjects for genes/regions important for FN bone geometry. The most significant gene identified in stage 1 was further studied in three additional unrelated Caucasian and Chinese Han samples (stage 2). All samples studied, in both stage 1 and stage 2, are independent. Table 1 presents basic characteristics of the studied samples, with detailed information as follows:

**Table 1:** Characteristics of the Study Subjects

	Stage 1: Caucasians	Stage 2: Caucasians	Stage 2: Chinese	Stage 2: Chinese	
				
	GWAS unrelated Caucasians	Unrelated bone geometry sample	Unrelated bone geometry sample	Hip fracture sample	
				
				Case	Control
Number	987	1488	2118	350	350
Sex ratio (M/F)	496/491	660/828	1119/999	124/226	173/177
Age (years)	50.35 (18.31)	54.97 (16.77)	28.17 (8.31)	69.35 (7.41)	69.54 (6.09)
Weight (kg)	80.14 (17.80)	80.93 (18.01)	57.09 (9.46)	59.15 (12.05)	59.61 (10.84)
Height (cm)	170.83 (9.74)	170.32 (9.77)	164.10 (7.68)	162.84 (8.31)	159.41 (9.20)
Buckling ratio	12.01 (2.74)	12.79 (2.63)	10.60 (1.82)	—	—
Cortical thickness (cm)	0.16 (0.03)	0.15 (0.03)	0.16 (0.02)	—	—

*Note:* Data presented are unadjusted means (SD).

#### Caucasian GWAS sample and independent Caucasian sample for within-ethnic group replication study

In stage 1, for the GWAS, 1,000 unrelated samples were identified from our established and expanding genetic repertoire currently containing more than 6000 subjects. In stage 2, 1488 unrelated samples were further selected from the remainder of our established samples. All the chosen subjects were unrelated U.S. Caucasians of European origin. Here, *unrelated* means there are no family links among other subjects in the sample. The subjects were recruited for genetic research by advertising. In the recruitment, individuals with chronic diseases and conditions that potentially might affect bone metabolism were excluded. These diseases/conditions included chronic disorders involving vital organs (e.g., heart, lung, liver, kidney, and brain), serious metabolic diseases (e.g., diabetes, hypo- and hyperparathyroidism, hyperthyroidism, etc.), other skeletal diseases (e.g., Paget disease, osteogenesis imperfecta, rheumatoid arthritis, etc.), chronic use of drugs affecting bone metabolism (e.g., hormone-replacement therapy, corticosteroid therapy, anticonvulsant drugs), and malnutrition conditions (e.g., chronic diarrhea, chronic ulcerative colitis, etc.). In addition, subjects taking anti-bone-resorptive or bone anabolic agents/drugs, such as bisphosphonates, were excluded from this study. The purpose of these exclusion criteria was to minimize the effects of environmental and therapeutic factors to influence the skeletal system. Minimizing the influence of these variables should provide for relative augmentation of the effects of genetic factors in our study sample, thereby improving our study design through increased statistical power for detecting these genetic factors.

Areal BMD (g/cm^2^) and region area (cm^2^) of FN were measured using Hologic machines (Hologic, Inc., Bedford, MA, USA). The machines were calibrated daily. The coefficient of variation (CV) values of the DXA measurements for FN BMD and region area were 1.87% and 1.97%, respectively.

Using DXA-derived FN BMD and region area, we estimated two FN geometric variables. The algorithm and the underlying assumptions regarding the geometry and structure of FN were elaborated earlier.(31,32) Briefly, the method assumes that (1) bone within the FN region has the configuration of a uniform circular cylinder, (2) 60% of the measured bone mass is cortical (i.e., *f_c_* = 0.6), and (3) the effective BMD in fully mineralized bone tissue is 1.05 g/cm^3^ (i.e., ρ_*m*_ = 1.05 g/cm^3^). All these assumptions were substantiated as good approximations.(31)

The estimated FN geometric variables are CT (cortical thickness) and BR (buckling ratio)—an index for bone structural stability. They are computed as follows:


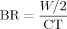



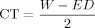


where


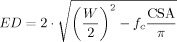


where *W* is the FN periosteal diameter and can be approximated by dividing the region area of FN by the width of the region of interest (in Hologic DXA systems, the width of the FN region is standardized at 1.5 cm).(21) CSA (cross-sectional area) is calculated as


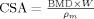


#### Chinese unrelated bone geometry sample

The 2118 unrelated Chinese subjects were healthy Chinese Han adults living in Changsha City. As with the Caucasian samples, Chinese subjects with chronic disorders involving vital organs, serious metabolic diseases, other skeletal diseases, chronic use of drugs affecting bone metabolism, and malnutrition conditions were excluded from the study. Areal BMD (g/cm^2^) and region area (cm^2^) of FN were measured using Hologic 4500W machines (Hologic, Inc., Bedford, MA, USA) under the same strict protocols used with the Caucasian sample. The CV of the DXA measurements for FN BMD and region area were 3.43% and 2.33%, respectively. BR and CT were estimated under the same method as those used for the Caucasians.

#### Chinese unrelated hip fracture sample

The Chinese unrelated hip fracture samples were recruited from Xi'an City or neighboring areas, which is more than 1000 km from Changsha City. It therefore was an independent Chinese cohort. The sample consisted of 350 patients with osteoporotic hip fracture (including 124 males and 226 females) and 350 healthy normal control individuals (including 173 males and 177 females). Affected individuals with low-trauma hip fracture were recruited between 2004 and 2007 from the affiliated hospitals and their associated clinics of Xi'an Jiaotong University. Inclusion criteria for cases were (1) age at hip fracture > 55 years for both male and female subjects and all women enrolled were postmenopausal, (2) minimal trauma fractures, usually owing to falls from standing height or less, (3) fracture site at FN or intertrochanter, and (4) hip fracture was identified through diagnosis of orthopedic surgeons/radiologists according to radiologic reports or X-rays. Patients with pathologic fractures, high-trauma fractures (such as motor vehicle accident), and late complications of fractures of the proximal femur requiring a second intervention were excluded. Control subjects were enrolled with the following inclusion criteria: (1) age at exam ≥ 60 years and (2) exclusion of subjects with chronic diseases and conditions that potentially might affect bone mass, structure, or metabolism (the exclusion criteria are the same as those adopted in recruitment of Caucasian subjects).

### Genotyping

#### Caucasian GWAS sample

Genomic DNA was extracted from whole human blood using a commercial isolation kit (Gentra Systems, Minneapolis, MN, USA) following the protocols detailed in the kit. Genotyping with the Affymetrix Mapping 250K Nsp and Affymetrix Mapping 250K Sty arrays was performed using the standard protocol recommended by the manufacturer. Fluorescence intensities were quantitated using Affymetrix Array Scanner 30007G. Data management and analyses were performed using the Affymetrix GeneChip Operating System. Genotyping calls were determined from the fluorescent intensities using the dynamic modeling (DM) algorithm with a 0.33 *P*-value setting,(33) as well as the Bayesian robust linear model with Mahalanobis distance (B-RLMM) algorithm.(34) DM calls were used for quality control, whereas the B-RLMM calls were used for all subsequent data analyses. B-RLMM clustering was performed with 94 samples per cluster.

Following an Affymetrix guideline, we set a standard for the minimum DM call rate at 93% for a sample, considering all the single-nucleotide polymorphisms (SNPs) in the two arrays, the 250K Nsp and 250K Sty arrays. Eventually, 997 subjects who had at least one array (Nsp or Sty) reaching a 93% call rate were retained. Because of missing data for bone geometry phenotypes among the 997 subjects, the effective sample size for the GWAS was 987 for both BR and CT. The average call rate for the 987 analyzed subjects reached greater than 95%. Of the initial full set of 500,568 SNPs, we discarded 32,961 SNPs with SNP-wise call rate greater than 95%, 36,965 SNPs with allele frequencies deviating from Hardy-Weinberg equilibrium (HWE, *P* < .001), and 51,323 SNPs with minor allele frequency (MAF) less than 1%. Therefore, the final SNP set for the GWAS scan contained 379,319 SNPs, yielding a genomic marker spacing of approximately 7.9 kb on average.

#### Caucasian bone geometry sample

Genomic DNA extraction and quality control procedures are the same as those used for the GWAS sample. Genotyping of the interesting SNPs identified from the GWAS in the Caucasian unrelated sample was performed by KBioscience (Herts, UK) using the technology of competitive allele-specific PCR (KASPar), which is detailed at the company's Web site (http://www.kbioscience.co.uk). The replication rate (duplicate concordance rate) was 99.7% for genotyping, and the average call rate was 97.8%.

#### Chinese bone geometry sample

Genomic DNA was isolated from whole blood using the phenol-chloroform extraction method. The concentration of DNA was quantified using a DU530 UV/VIS Spectrophotometer (Beckman Instruments, Fullerton, CA, USA). The same set of SNPs used for the Caucasian bone geometry sample was successfully genotyped in the Chinese bone geometry sample of 2118 subjects. Genotyping was performed using a primer extension method with MALDI-TOF mass spectrometry for multiplexed genotyping of SNPs on a MassARRAY system, as suggested by the manufacturer (Sequenom, Inc., San Diego, CA, USA) and described by Braun and colleagues.(35) The replication rate was 99.2%, and the average call rate was 97.2%.

#### Chinese hip fracture sample

Genomic DNA extraction methodology was the same as that used with the Chinese bone geometry sample. The 350 hip fracture cases and 350 controls were genotyped using Affymetrix Mapping 250K Nsp and Affymetrix Mapping 250K Sty arrays. The final mean B-RLMM call rate reached a level of 99.02%, and the duplication rate was 99.70%. The discordant genotypes were discarded. Genotypes of interesting SNPs in this cohort were obtained from the Affymetrix 500K chips.

### Statistical analyses

In all four group samples (i.e., Caucasian GWAS, Caucasian bone geometry, Chinese bone geometry, and Chinese hip fracture cohorts), HWE was assessed by chi-square analyses. SNPs that did not follow HWE were excluded from further data analyses.

For the stage 1 Caucasian GWAS sample, parameters such as age, age^2^, sex, age/age^2^-by-sex interaction, height, and weight were tested for their associations with BR and CT using stepwise regression. Significant (*P* ≤ .05) terms were included as covariates to adjust the raw BR and CT values for subsequent analyses. For BR, the covariates were age, height, weight, sex, and age^2^ × sex. For CT, the covariates were age, weight, and age^2^. The residuals from a linear model after adjusting for the significant covariates were used as traits in the follow-up data analyses. To minimize possible spurious associations owing to potential population stratification, we used EIGENSTRAT(36) software in GWAS association analyses. EIGENSTRAT detects and corrects for ancestry information while performing association analyses between phenotypes and genotypes. We used EIGENSTRAT because it can appropriately handle the quantitative data while maintaining sufficient power and robustness.(36) In the data analyses, the SNPs were coded as 0, 1, and 2 to represent AA, AB, and BB genotypes.

Multiple testing is a perplexing issue in GWAS. Because the Bonferroni correction is considered overly conservative given extensive linkage disequilibrium (LD) among markers, we adopted the GWAS significance threshold of approximately 4.2 × 10^−7^ proposed by Lencz and colleagues.(37) The gene-wise approach used to calculate this threshold took into account recent estimates of the total number of genes in the human genome.

The LD patterns of the most significant gene were analyzed and plotted using the Haploview program(38) (http://www.broad.mit.edu/mpg/ haploview/). Focused association analyses on certain SNPs and other miscellaneous statistical analyses were performed using software packages SAS (SAS Institute, Inc., Cary, NC, USA) and Minitab (Minitab, Inc., State College, PA, USA), which include descriptive statistics data analysis and multiple regression analysis to screen significant covariates for BR and CT and the normality of the adjusted BR and CT data.

For stage 2 replication analyses of the significant gene, significant parameters (*P* < .05) such as age, age^2^, sex, age/age^2^-by-sex interaction, height, and weight were used as covariates to adjust for the raw BR and CT values. We used a stepwise regression model to screen significant covariates for each study cohort. Since the effects of these confounding factors on the traits were different in each cohort, different covariates were applied. For unrelated bone geometry Caucasian samples (*n* = 1488), the covariates were age, sex, age × sex, weight, and height for BR; weight, age^2^, age^2^ × sex, and age × sex for CT. For unrelated Chinese bone geometry samples (*n* = 2118), the covariates were age^2^, weight, height, and age^2^ × sex for BR and weight, age × sex, age, and sex for CT. The residuals from a linear regression model after adjusting for the significant covariates were used as traits in the in HelixTree (http://www.goldenhelix.com/SNP_Variation/HelixTree/index.html). The SNPs were coded numerically with 0 for AA, 1 for AB, and 2 for BB, respectively. In the replication stage, in order to account for the multiple-testing problem, the SNPSpD method(39) (http://gump.qimr.edu.au/general/daleN/SNPSpD/) was adopted to infer the effective number of independent tests (*M*eff), and the overall significance level was set at 0.05/*M*eff.

The *P* values from cohorts of Chinese and Caucasian samples for bone geometry were combined using Fisher's method(40) to quantify the overall evidence for association with bone geometry variation. No cohort/geography adjustment was performed because the tests for Caucasian and Chinese are independent and have the same null hypotheses. In the Chinese hip fracture sample, genotype distributions for SNPs were compared using logistic regression models controlling for sex, age, height, and weight as covariates between the fracture versus nonfracture pooling sample including both males and females.

To detect population stratification that may lead to spurious association results, we used the software Structure 2.2 (http://pritch.bsd.uchicago.edu/software.html) to investigate the potential substructure/stratification of our sample. The Structure 2.2 program uses a Markov chain Monte Carlo (MCMC) algorithm to cluster individuals into different cryptic subpopulations on the basis of multilocus genotype data.(41) Using the software, we performed independent analyses under an assumption of *K* = 2 population strata using 200 unlinked markers in the GWAS unrelated Caucasian cohort and 1000 unlinked markers in the Chinese hip fracture cohort.

## Results

### GWAS in the Caucasian sample

**Fig. 1:**
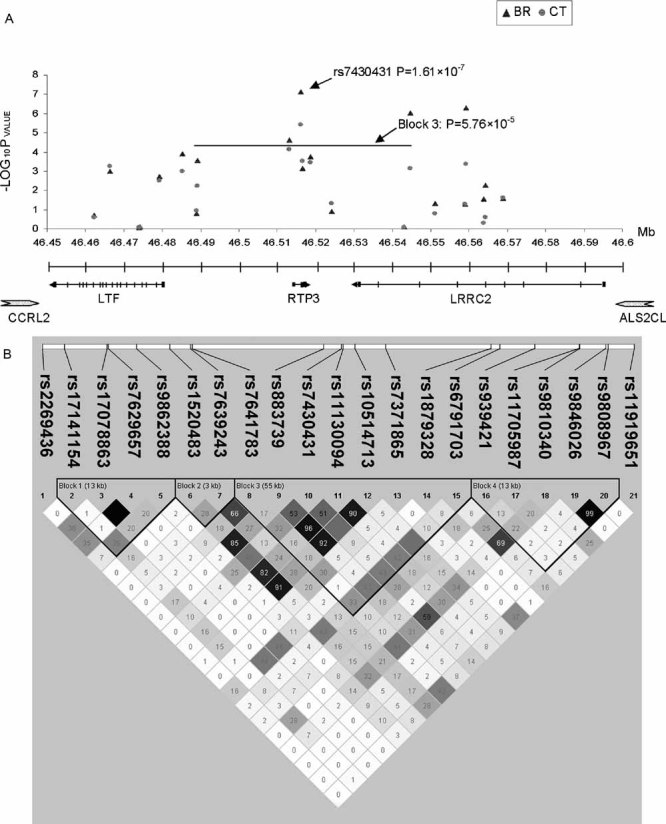
Schematic view of association results, marker density, and LD structure within and adjacent to the *RTP3* gene. (*A*) Genome-wide association study (GWAS) results for both single-marker and LD-block haplotype (*solid line*) association results. The SNP rs7431430 achieved the most significant association with BR (*P* = 1.61 × 10^−7^). Block 3 achieved a highly suggestive association with BR (*P* = 5.76 × 10^−5^). *RTP3* = receptor (chemosensory) transporter protein 3; *LRRC2* = leucine-rich repeat containing 2; *LTF* = lactotransferrin; *CCRL2* = chemokine (C-C motif) receptor-like 2; *ALS2CL* = ALS2 C-terminal like. (*B*) Gene structure and LD patterns for the 21 SNPs within or adjacent to the *RTP3* gene in GWAS Caucasian samples. Exons are depicted with a vertical bar. Positions of the 21 SNPs (listed in Table 2) are sketched. LD block structure, as depicted by Haploview, is shown in the bottom frame. The increasing degree of darkness from white to black represents the increasing strength of LD (measured by *r*^2^).

**Fig. 2:**
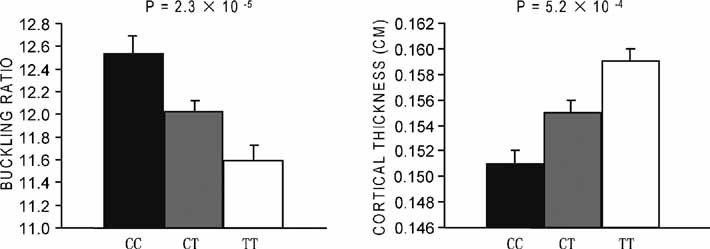
Least square means (± SE) of BR and CT values in the three genotypes of SNP rs7430431 in the unrelated GWAS Caucasian subjects. A linear regression model was used with age, age^2^, sex, age/age^2^-by-sex interaction, height, and weight as covariates. *P* values of one-way ANOVA for testing the genotype effects on adjusted BR and CT are given.

Using EIGENSTRAT, which controls for potential population stratification, we performed a genotype association analysis. For all the analyzed SNPs and bone geometry phenotypes, we observed one SNP (rs7430431) beyond the GWAS significant threshold for BR (*P* = 1.61 × 10^−7^; Table 2). This SNP also was found to have a highly suggestive association with FN CT (*P* = 1.92 × 10^−6^). SNP rs7430431 is located in 3p21.31, a region that we previously found to be linked with CT (LOD = 2.19, *P* = .0006) in 3998 individuals from 434 pedigrees.(16) The minor allele frequency (MAF) of rs7430431 was 49.4% according to our data and 48.3% according to the HapMap CEU database (see Table 2). The SNP was in HWE and located in the intron of the *RTP3* gene. The *RTP3* gene, which has a total length of approximately 3 kb, contains two exons and one intron. In this 3-kb region, a total of eight SNPs were genotyped. Besides rs7430431, most other SNPs in the region also achieved highly suggestive association with both BR and CT (*P* ≤ .001; see Table 2). We selected 21 SNPs (see Table 2) within and adjacent to *RTP3* to reconstruct the LD block using Haploview in order to further study this gene. Four blocks were reconstructed (Fig. 1). Haplotype data analysis indicated that haplotype block 3, containing rs7430431 has a highly suggestive association with BR (*P* = 5.76 × 10^−5^) (see Fig. 1). We also compared adjusted BR and CT values between different genotypes of SNP rs7430431. In our GWAS subjects, as shown in Fig. 2, subjects with the CC genotype had the highest BR [12.54 ± 0.15 (±SE)] and the lowest CT (0.151 ± 0.001 cm), individual of CT genotype had intermediate BR (12.02 ± 0.10) and CT (0.155 ± 0.001 cm), and individuals of the TT genotype had the lowest BR (11.59 ± 0.14) and the highest CT (0.159 ± 0.001 cm). Thus rs7430431 genotypic effects on BR and CT are approximately additive (Fig. 2). In our study subjects, individuals with the CC genotype had, respectively, approximately 8.2% higher BR and 5.0% lower CT than those with the TT genotype, which consistently leads to poor femoral neck bone geometry.

**Table 2:** Information for the 21 Analyzed SNPs Within and Adjacent to the *RTP3* Gene and Their Association Results in GWAS Analysis Using EIGENSTRAT

SNP ID	Name	Position	Gene	Role	Allele^a^	MAF^b^	MAF^c^	*P* value for BR	*P* value for CT
1	rs2269436	46462257	*LTF*	Intron	G/A	0.042	0.042	2.38 × 10^−1^	2.87 × 10^−1^
2	rs17141154	46462271	*LTF*	Intron	A/G	0.167	0.167	1.74 × 10^−3^	6.21 × 10^−4^
3	rs17078863	46473920	*LTF*	Intron	C/T	0.045	0.042	5.02 × 10^−1^	3.05 × 10^−1^
4	rs7629657	46474176	*LTF*	Intron	T/C	0.048	0.042	4.89 × 10^−1^	2.95 × 10^−1^
5	rs9862388	46479320	*LTF*	Intron	G/A	0.300	0.267	2.38 × 10^−1^	2.87 × 10^−1^
6	rs1520483	46485213	*RTP3*	Promoter	T/C	0.350	0.374	1.81 × 10^−4^	7.14 × 10^−4^
7	rs7639243	46488981	*LTF*	Promoter	A/C	0.400	0.333	2.32 × 10^−1^	2.21 × 10^−1^
8	rs7641783	46489276	*RTP3*	Promoter	T/C	0.273	0.258	4.06 × 10^−4^	6.56 × 10^−5^
9	rs883739	46513022	*RTP3*	Promoter	A/C	0.352	0.375	4.99 × 10^−5^	8.87 × 10^−5^
10	rs7430431	46516151	*RTP3*	Intron	C/T	0.494	0.483	**1.61 ×** **10**^−**7**^	1.92 × 10^−6^
11	rs11130094	46516507	*RTP3*	Intron	A/G	0.404	0.364	1.03 × 10^−3^	2.49 × 10^−4^
12	rs10514713	46518621	*RTP3*	3′ UTR	A/C	0.380	0.381	2.68 × 10^−4^	3.09 × 10^−4^
13	rs7371865	46524192	*RTP3*	3′ UTR	A/C	0.105	0.075	2.49 × 10^−1^	1.93 × 10^−1^
14	rs1879328	46543161	*LRRC2*	Intron	G/A	0.167	0.140	8.10 × 10^−1^	8.40 × 10^−1^
15	rs6791703	46544725	*LRRC2*	Intron	G/T	0.498	0.500	8.69 × 10^−7^	1.80 × 10^−4^
16	rs939421	46551085	*LRRC2*	Intron	G/A	0.300	0.225	4.90 × 10^−2^	1.60 × 10^−1^
17	rs11705987	46559076	*LRRC2*	Intron	C/A	0.210	0.246	7.54 × 10^−2^	5.40 × 10^−2^
18	rs9810340	46559176	*LRRC2*	Intron	T/A	0.450	0.458	5.33 × 10^−7^	7.70 × 10^−5^
19	rs9846026	46563817	*LRRC2*	Intron	A/C	0.100	0.078	2.86 × 10^−2^	3.90 × 10^−1^
20	rs9808967	46564298	*LRRC2*	Intron	T/A	0.050	0.076	3.03 × 10^−3^	9.17 × 10^−2^
21	rs11919651	46568910	*LRRC2*	Intron	C/A	0.180	0.158	3.71 × 10^−2^	4.60 × 10^−2^

*Note:* The RTP3 gene is located in 3p21.31. MAF = minor allele frequency; BR = buckling ratio; CT = cortical thickness.

a The former allele represents the minor allele of each locus.

b Minor allele frequency calculated in our own Caucasian sample.

c Minor allele frequency reported for Caucasians in the public database such as Hap Map or dbSNP.

d *P* values were calculated using EIGENSTRAT analyses. Significant results at the GWAS level are labeled in bold.

### Replication studies in independent Caucasian and Chinese samples

Replication studies confirmed the association of the *RTP3* gene to BR, CT, and hip fracture (Table 3). According to the HapMap-HCB (Han Chinese in Beijing) database, the MAF of SNP rs7430431 is less than 5% (4.4%), indicating a significant MAF difference between Chinese and Caucasian samples. In addition to rs7430431, SNP rs10514713 was selected for further analysis in order to ensure high heterozygosity of the SNPs for replication studies and therefore increase statistical power. SNP rs10514713 is in high LD with rs7430431 and is located in the 3′UTR of the *RTP3* gene. There was evidence for association between rs7430431 and both BR (*P* = .020) and CT (*P* = .020) in an independent sample of 1488 Caucasians. An association also was detected between BR and rs7430431 (*P* = .040) and rs10514713 (*P* = .049) (see Table 3) in 2118 unrelated Chinese. A combined association analysis using Fisher's method showed that rs7430431 was significantly associated with both BR (*P* = .007) and CT (*P* = .014) (see Table 3). Further, the direction of this association is the same as that in the original GWAS Caucasian sample; i.e., C-allele carriers had higher BR and lower CT values than T-allele carriers.

**Table 3:** Replication Association Results in Stage 2

dbSNP	rs7430431	rs10514713
Role	Intron	3′ UTR
Alleles	C/T	A/C
*Unrelated bone geometry Caucasian sample (1488)*		
MAF	.409	.335
*P* value for BR	**.020**	.135
*P* value for CT	**.020**	.198
*Unrelated Chinese bone geometry sample (2118)*		
MAF	.026	.486
*P* value for BR	**.040**	**.049**
*P* value for CT	.098	.093
*Combined Chinese and Caucasians (3606)*		
*P* value for BR	**.007**	**.040**
*P* value for CT	**.014**	.092
*Unrelated Chinese hip fracture sample (700)*		
MAF	.020	.429
*P* value for hip fracture	0.308	**.001**

*Note:* Stage 2 involved a total of 3606 subjects for replication analysis for BR and CT and 700 subjects for analysis for hip fracture. There is no overlap between these samples. *P* values were one-tailed. The combined *P* values were obtained based on Fisher's method.(40) No cohort/geography is adjusted. The results with *P* < .05 are in bold. MAF = minor allele frequency; BR = buckling ratio; CT = cortical thickness.

We tested two SNPs (SNPs rs10514713 and rs7430431) and two phenotypes (BR and CT) in our replication study. These tests resulted in a multiple-testing problem. In order to adjust for multiple testing, we used the SNPSpD method to set the overall significance level. The *M*eff for BR and CT phenotypes is 1.357, and the *M*eff for the two tested SNPs (SNPs rs10514713 and rs7430431) is 1.515. Thus the significant threshold for the replication stage is set at *P* = .05/(1.357 × 1.515) = 0.024. According to the threshold, most of our nominal significant results (*P* < .05) in Table 3 survive.

To further assess the relevance of the *RTP3* gene to hip fracture risk, we tested the association of the two SNPs with osteoporotic hip fracture in a Chinese sample consisting of 350 hip fracture patients and 350 healthy controls. A significant association was found between SNP rs10514713 (MAF = 42.9%) and hip fracture (*P* = .001; see Table 3).

## Discussion

Our GWAS and follow-up validation/replication studies in independent Caucasian and Chinese samples identified *RTP3* as a potential novel gene associated with variation of BR and CT at the FN. BR and CT are geometric variables associated with hip fracture.(31,32,42) BR is an index for bone structural instability(31,43,44) and is positively associated with hip fracture. CT is proportional to volumetric and areal BMD with the assumption that the bone within the FN region is a uniform circular cylinder. CT is negatively correlated with hip fracture risk.(31,42) These two geometric measures are highly correlated with one another and have been reported as important predictors of the incidence of hip fracture.(12,31,45–47) In addition to BR and CT, in the current GWAS of 987 unrelated Caucasian subjects using 379,000 SNPs, we also tested other bone geometric measures, such as CSA and section modulus. However, none of the SNPs associated with any known genes reached the GWAS significance threshold (4.2 × 10^−7^). Since four FN geometry phenotypes are tested in the data analyses and our threshold (4.2 × 10^−7^) may need to be adjusted. We did not adopt a more stringent threshold for the following reasons: (1) We are reporting the most significant results, and the results have been replicated in other independent cohorts, especially in hip fracture cohorts. A stringent GWAS threshold will not change our results. (2) The four phenotypes are highly correlated, and a Bonferroni correction will be too conservative for the study.

It should be noted that bone geometry phenotypes are dependent on body size and shape.(48) In our study, although height and weight were used as covariates to adjust size variation, the adjustment cannot totally remove the body size and body shape effect. Currently, there is no established appropriate method to correct body size and body shape effect.

The powerful GWAS analyses reported here identified an intronic polymorphism (rs7430431) in the *RTP3* gene as an SNP that was significantly associated with BR variations. Since rs7430431 has a low MAF in Chinese samples, we tested rs7430431 plus a nearby common SNP of the *RTP3* gene in our replication studies with independent Caucasian and Chinese samples. These replication analyses confirmed the association between the *RTP3* gene and BR. Importantly, the relevance of the *RTP3* gene to hip fracture risk also was detected in an independent Chinese sample containing 700 subjects. Since association results may be biased by population stratification, we analyzed for potential population stratification for the cohorts using software Structure 2.2(41) in a GWAS unrelated Caucasian sample and hip fracture sample. All subjects in the two cohorts were tightly clustered together, suggesting no population stratification.

The *RTP3* gene is located at 3p21. This genomic region was reported to be linked with CT (LOD = 2.19, *P* = .0006) in our prior linkage study performed in 3998 individuals from large pedigrees.(16) CT is an estimate with an assumption that a 60% of cortical mass is uniformly distributed in the femoral neck region. BMD is an estimate of bone mineral uniformly distributed in a region of interest. Since CT and BMD are highly correlated, we also checked the importance of the 3p21 region for BMD variation. A recent meta-analysis of BMD linkages(49) failed to identify this region as an important quantitative trait locus (QTL). However, some independent linkage studies support the concept that 3p21 is an important region for FN and spine BMD variations.(50–53) In 155 osteoporotic families containing 654 study individuals, Duncan and colleagues reported that the 3p21 region achieved a suggestive LOD score for FN BMD (LOD = 2.7).(50) In addition, a study involving nonidentical twin pairs (in 1094 pedigrees) detected a suggestive LOD score of 2.7 at chromosome 3p21 for spine BMD.(52)

We referred to other reported GWAS in the bone field to look for support for the association of the *RTP3* gene and FN bone geometry. Among them,(28–30,54) FN bone geometry results are only reported in the Framingham study,(30) which provides strong, independent support for our finding.(30) In the National Center for Biotechnology Information (NCBI) dbGaP database (http://www.ncbi.nlm.nih.gov/sites/entrez?db=gap), two SNPs (SNP9 rs883739 and SNP12 rs10514713 in Table 2) in the *RTP3* gene, which are in high LD with rs7430431 (*r*^2^ = 0.641 between SNP9 rs883739 and SNP10 rs7430431 and *r*^2^ = 0.638 between SNP12 rs10514713 and SNP10 rs7430431 in HapMap-CEPH), were genotyped in the Framingham 100,000 GWAS. In the Framingham study, both SNP9 rs883739 and SNP12 rs10514713 were significantly associated with FN BR (*P* = .030 and *P* = .017, respectively) in women.

*RTP3* is a novel gene first identified in 2002. Several lines of evidence support the concept that *RTP3* may functions as a tumor suppressor gene.(55–57) In addition, the *RTP3* protein product shares homology with other *RTP* family members, which have been shown to contribute to functional cell surface expression of olfactory receptors on olfactory neurons. However, in contrast to *RTP1* and *RTP2*, *RTP3* is not expressed in olfactory neurons. Therefore, it appears unlikely that *RTP3* influences olfaction in a manner similar to that described for other members of the *RTP* family.(58) It is unknown whether *RTP3* has other biologic functions.

This is the first study reporting the potential importance of the *RTP3* gene to femoral neck bone geometry. However, the mechanism by which it contributes to femoral neck bone geometry and osteoporotic fracture risk is unknown. It has been suggested that *RTP3* may interact with *FHIT* (fragile histidine triad gene), which downregulates the *SMAD4* (SMAD family member 4) gene.(59–62) *SMAD4* is a transcription corepressor for the *ESR1* (estrogen receptor alpha) gene and strongly inhibits the transcription of various downstream genes of estrogen signaling.(63) Further, it has been shown that *SMAD4* has direct physical interactions with *ESR1* and serves as a common signal transducer in the bone morphogenetic protein (BMP)/transforming growth factor-beta (TGF-β) signaling pathway.(64,65) Both estrogen signaling and BMP/TGF-β signaling pathways are important for bone development and skeleton morphogenesis.(66,67) Furthermore, *SMAD4* may directly regulate endochondral bone formation,(68) cartilage growth,(69) bone healing,(70) osteogenesis,(71) and long bone development.(72) Taken together, our study suggests that *RTP3* may be a novel candidate gene influencing bone geometry variation, potentially via *FHIT* and *SMAD4*.

Several limitations of our studies need to be mentioned. First, the unrelated Chinese bone geometry sample was much younger than the discovery cohort and other replication samples. In our study, although age was used as a covariate to adjust the raw BR and CT values, the adjustment may not totally eliminate the age-related difference between groups. Therefore, our replication result may suggest that the *RTP3* gene is important for femoral neck bone geometry in both young and old population. Second, different genotyping methods were used for different replication studies owing to different genotyping platforms across the cooperative laboratories. Since all the genotyping methods had high call and replication rates, disparity of the genotyping methods should be acceptable. In actuality, it is common to see the issue of varying genotyping methods arise in GWAS replication studies. For example, Luciano and colleagues used KASPar, MALDI-TOF, and Taqman genotyping methods in their replication studies.(73) With the development of genotyping technology, the concordant rate among different genotyping methods (including MALDI-TOF and KASPar) can be as high as 94% and even greater than 99.9%.(74,75) Third, although the most significant SNP (rs7430431) was consistently associated with BR in all bone geometry replication studies, no evidence of association was detected between rs7430431 and hip fracture. This may be attributable to the low MAF (MAF = 0.02) of this SNP in the Chinese hip fracture sample. The significant association of a nearby common SNP (rs10514713) (MAF = 42.9%) with hip fracture (*P* = .001) provides supportive evidence for the potential importance of the *RTP3* gene to the risk of hip fracture. Fourth, although SNPs rs10514713 and rs7430431 are tested to be important for bone geometry variation, without a functional study, it is difficult to assert their importance. Without further functional study, it is also difficult for us to confirm the importance of *RTP3* in bone development at early ontogenesis and its outcomes later in the life. Fifth, our bone geometry estimates, based on the earlier work,(21,31,32,76) relied on the assumptions of FN shape as a uniform circular cylinder. This assumption may not be completely realistic.(32)

In summary, for the first time, our GWAS identified a novel gene, *RTP3*, significantly associated with bone femoral geometry. The gene's relevance to hip fractures and femoral neck bone geometry was further replicated and confirmed in independent Caucasian and Chinese samples. Our findings suggest that *RTP3* may be a promising candidate gene for osteoporotic fractures in different ethnic groups. Various further analyses, such as replication in other ethnic populations and molecular functional studies, are needed to validate this finding and delineate the mechanism by which this gene influences femoral neck bone geometry and fracture risk.
